# Administration of Perchloride of Iron

**Published:** 1873

**Authors:** 


THE ADMINISTRATION OF PERCHLORIDE OF IRON.—Delicate patients, says Dr. Herbert Snow, very frequently object to the astringent metallic taste long remaining in the mouth after the administration of tincture of perchloride of iron, the flavor of which is but very imperfectly disguised by the syrup or spirit of chloroform with which it is usually ordered. It is worth knowing that the substitution of a small quantity of glycerine, about half an ounce to an eight ounce mixture, will altogether obviate this inconvenience.—*British Medical Journal.*

